# First fluconazole-resistant *Candida auris* isolated from fungal otitis in Iran

**DOI:** 10.18502/cmm.7.1.6243

**Published:** 2021-03

**Authors:** Mojtaba Taghizadeh Armaki, Saeid Mahdavi Omran, Keyvan Kiakojuri, Shaghayegh Khojasteh, Jalal Jafarzadeh, Mahin Tavakoli, Hamid Badali, Iman Haghani, Tahereh Shokohi, Mohammad Taghi Hedayati, Mahdi Abastabar

**Affiliations:** 1 Department of Medical Mycology and Parasitology, School of Medicine, Babol University of Medical Sciences, Babol, Iran; 2 Department of Ear, Nose, and Throat, Faculty of Medicine, Roohani Hospital, Babol University of Medical Sciences, Babol, Iran; 3 Invasive Fungi Research Center, Communicable Diseases Institute, Mazandaran University of Medical Sciences, Sari, Iran; 4 Department of Medical Mycology, School of Medicine, Mazandaran University of Medical Sciences, Sari, Iran; 5 Student Research Committee, Mazandaran University of Medical Sciences, Sari, Iran

**Keywords:** *Candida auris*, Fluconazole-resistant, Iran

## Abstract

**Background and Purpose:**

*Candida auris*, as a new characterized pathogenic yeast, has attracted remarkable attention in the recent decade due to its rapid global emergence and multidrug resistance traits. This unique species is able to cause nosocomial outbreaks and tolerate adverse conditions; however, it has been mostly misidentified by conventional methods.

**Case report:**

This report aimed to describe the first fluconazole-resistant case of *C. auris* otitis in an immunocompetent patient in Iran. The isolate showed minimum inhibitory concentration of ≥ 32 μg/ml for fluconazole; however, the patient was treated with topical clotrimazole and miconazole with no recurrence.

**Conclusion:**

This was the second strain of *C. auris* isolated from otitis in Iran which was fluconazole-resistant, unlike the first Iranian isolate.

## Introduction

After the first report about the ear canal involvement in a Japanese patient in 2009, *Candida auris* has been identified in all continents and nearly 40 countries [ 1 ]. It has generally been isolated from the blood, ear, skin, central nervous system, bone, respiratory tracts, wounds, axilla, urine, bile, nares, esophageal mucosa, and the rectum [ 2 ]. This new fungal pathogen is known as a serious concern for human health due to its biofilm formation ability, range of virulence factors, mis-identification, the ability of long-term survival on surfaces and horizontal spread in hospitals, association with hospital outbreaks, and high mortality rates in debilitated patients residing in intensive care units.

Until now, outbreaks due to *C. auris* have been reported in countries, such as the USA, the UK, Venezuela, Pakistan, and Colombia [ 3 ]. Moreover, a great proportion of *C. auris* strains present decreased sensitivity or resistance to multiple and even to all antifungals [ 4 ]. The *C. auris* in Iran was first identified in 2018 in the external ear canal discharge of an immunocompetent female with a history of otalgia, itching, hearing loss, and tympanic membrane perforation (TMP) [ 5 ]. This isolate was susceptible to fluconazole with a minimum inhibitory concentration (MIC) value of 16 μg/mL and also other triazoles. Herein, we report the first fluconazole-resistant *C. auris* strain in a patient with chronic mycotic otitis externa in Iran.

## Case report

In August 2020, a 40-year-old female, with a three-year history of bilateral otalgia with severe itching and creamy discharge from the ear canal was admitted to the department of otorhinolaryngology. She complained of ear eczema and received ciprofloxacin and gentamicin for possible bacterial infections. She also received betamethasone for inflammation (i.e., redness and swelling) and itching of the external ear canal. Notably, she had no history of traveling abroad, and the only predisposing factor which the patient remembered was repeated ear manipulation.

**Figure 1:**
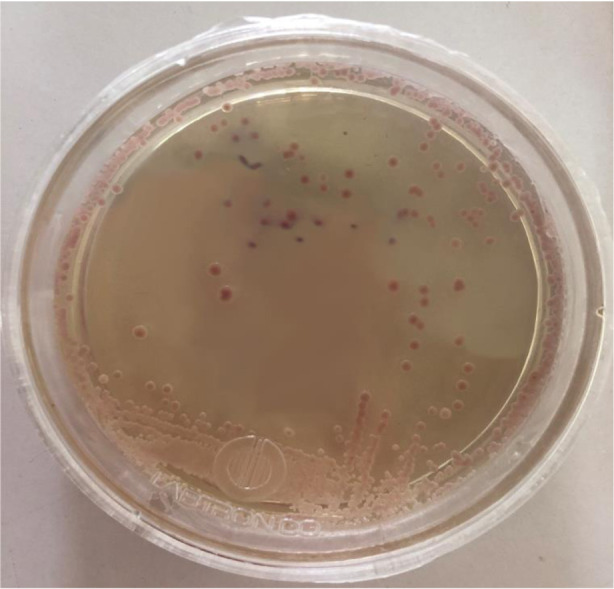
Pure culture of isolate on CHROMagar Candida (pink color) incubated at 30 °C for 48 h

The ear discharge was collected using a cotton swab and inoculated on Sabouraud dextrose agar (Difco, USA) supplemented with chloramphenicol and CHROMagar *Candida* (CHROMagar Company, France). It is noteworthy that round to ovoid yeast cells were observed in the direct examination. After incubation for 48 h at 30 °C, it was identified initially as non-*albicans* *Candida* due to the growth of pale pink to dark purple colonies on CHROMagar *Candida* (Figure 1). Identification was performed by amplification of internal transcribed spacer (ITS) regions using ITS1 and ITS4 as described previously [ 5 , 6 ].

**Figure 2:**
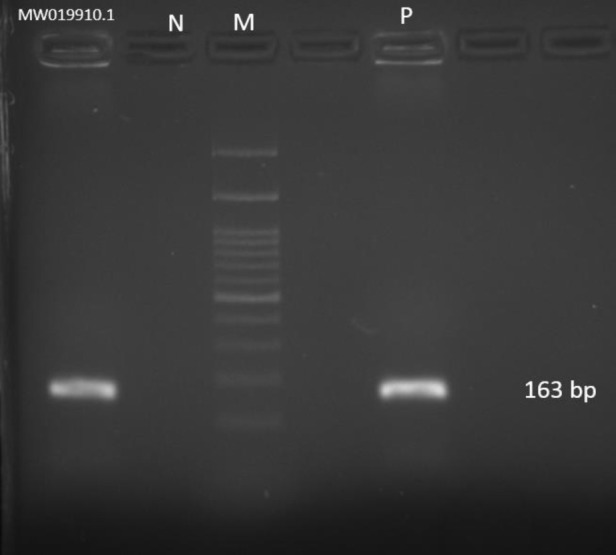
Gel electrophoresis of *C. auris*-specific polymerase chain reaction product analysis. M, 100-bp DNA ladder Lane 1, *C. auris* (MW019910.1), N, negative control; P, positive control *C. auris* (MK123931.2)

**Figure 3:**
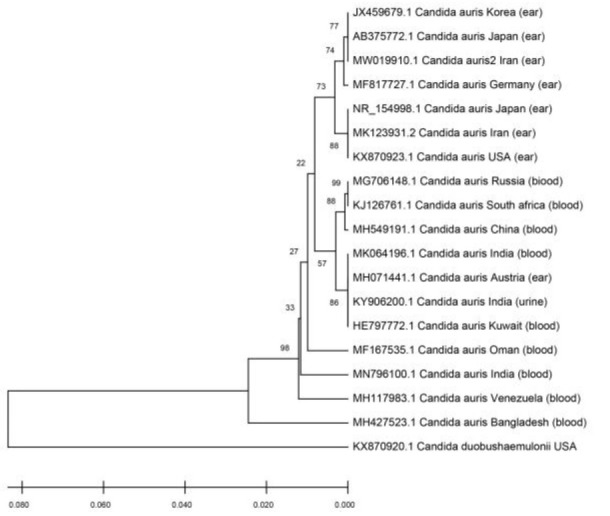
Phylogenetic tree generated by unweighted pair group method with arithmetic mean analysis using ITS sequences of the *C. auris* strains with closely related *Candida* species

The DNA sequences of the ITS region were analyzed using GenBank, and the Basic Local Alignment Search Tool showed 100% similarity with the ex-type isolate of *C. auris* (MH427523) and deposited with the accession number of MW019910.1. Moreover, polymerase chain reaction (PCR) was performed using specific primers for *C. auris* designed by Kordalewska et al. [ 7 ] which resulted in a 163-bp PCR product (Figure. 2) The unweighted pair group method with arithmetic mean analysis was performed in MEGA X software (version 10.1) with bootstrapping using 1000 replicates (Figure 3).

The in vitro antifungal susceptibility testing was performed on fluconazole, voriconazole, amphotericin B, nystatin, miconazole, itraconazole, posaconazole, ravuconazole, isavuconazole, ketoconazole, and anidu-lafungin using the CLSI M27-S4 broth microdilution document [ 7 ]. Based on the results of the antifungal susceptibility test, *C. auris* isolate had a high fluconazole MIC (≥32 µg/ml). The MIC values of voriconazole, itraconazole, miconazole, amphotericin B, posaconazole, isavuconazole, ravuconazole, ketoconazole, clotrimazole, nystatin, micafungin, and anidulafungin were 1, 0.016, > 16, 0.25, 0.016, 0.016, 0.5, 2, 2, 2, 0.032, and 0.016 μg/mL, respectively. 

The otomycosis was managed by antifungal therapy consisting of topical administration of clotrimazole 1% and miconazole 1% cream b.i.d. for 

eight weeks. During the follow-up, it was found that the patient had completely improved, and there was no evidence of recurrence.

This study was approved by the Ethics Committee of the Babol University of Medical Science (ethics code: IR.MUBABOL.REC.1399.288). This case report was performed in accordance with the Declaration of Helsinki, and informed consent was obtained from the patient for the inclusion of the details in the manuscript and for publication.

## Discussion

To date, more than 4000 cases of *C. auris* infection and colonization have been recorded and they have been rising over the past several years radically [ 9 ]. Notably, the most frequently reported type of invasive infection of *C. auris* is bloodstream infection which often leads to high mortality rates (30-60%) [ 10 , 11 ]. Due to the intrinsic resistance of *C. auris* to one or more classes of antifungal agents, it is considered as a “superbug”. 

In addition, according to previous studies, 90% of *C. auris* strains are resistant to fluconazole (MIC ≥ 32 μg/mll). High MIC values for amphotericin B were presented in 10–30% of *C. auris* isolates, and < 5% of isolates were resistant to echinocandins [ 7 , 12 ].

The current report is the second report of Iranian *C. auris* causing fungal otitis externa and also the first fluconazole-resistant strain of this species. In 2018, we reported the first isolate of *C. auris* responsible for otomycosis in a healthy 14-year-old Iranian female with only frequent swimming as the predisposing factor and TMP as the most serious clinical presentation [ 5 ]. The present case occurred in a female with eczema and ear manipulation as the most important predisposing factors with no history of travel outside Iran. 

Subsequent to the initial reports of ear infections of *C. auris* in Japan and Korea in 2009 [ 12 , 13 ], this species was also isolated from otitis samples in some other countries, including Austria [ 15 ], Switzerland [ 16 ], USA [ 17 ], Canada [ 18 ] and Pakistan [ 19 ]. The second Japanese *C. auris* strain was isolated from the tympanic cavity discharge in 2018 by Iguchi et al. [ 20 ] in which the MICs of fluconazole, micafungin, and amphotericin B were 4, 0.06, and 0.25 µg/mL, respectively [ 20 ]. The current isolate demonstrated high fluconazole MIC value (≥32 µg/ml), while the first Iranian strain had a MIC of 16 μg/ml for fluconazole. Recently, Alfouzan et al. [ 21 ] have declared that 100% of the strains of *C. auris* isolated from patients in Middle-Eastern countries are resistant to fluconazole. 

Majority of the reports have noted that *C. auris* isolates substantially show resistance against fluconazole while a lower proportion of them are resistant to amphotericin B and echinocandins, such as micafungin, and some of them displayed resistance against multiple antifungals [ 4 ]. Chowdhary et al. noted that 90%, 8%, 2.3%, and 2% of *C. auris* were resistant to fluconazole, amphotericin B, voriconazole, and echinocandins, respectively [ 22 ]. Pekard-Amenitsch et al. [ 23 ] isolated a susceptible *C. auris* isolate from an auditory canal in Austria which had good sensitivity to antifungal agents, including triazoles, polyene, and echinocandins.

The current isolate demonstrated low MICs against echinocandins, including micafungin and anidulafungin. Similarly, Lepak et al. [ 24 ] found that micafungin had the highest efficacy in comparison to fluconazole and amphotericin B in an animal model of *C. auris* candidemia. According to the results of phylogenetic analysis based on ITS rDNA, the second Iranian isolate of *C. auris* was nested within the group of *C. auris* ear isolates originating from Korea, Japan, the USA, and Germany. Furthermore, it was found to be more closely related to the previous Iranian strain. Despite using whole-genome sequencing of Iranian first isolate of *C. auris*, we found a potential fifth (V) clade for *C. auris* isolate alongside South Asia Clade (I), the East Asia Clade (II), the South Africa Clade (III), and the South America (IV) Clades [ 25 ].

In summary, the first and second Iranian isolates fall within the clade composed of *C. auris* ear isolates and were distinct from isolates of invasive infections and clonal outbreaks; however, the second strain was fluconazole-resistant which is a matter of further concern in the future.

## Conclusion

This report can encourage otorhinolaryngologists to pay more attention to the possibility of otitis externa caused by *C. auris* as a significant multidrug-resistant species. It is recommended to apply *C. auris*-specific primers for rapid identification of this unique species, and antifungal susceptibility test for the suspected cases with treatment failure.

## Authors’ contribution

M. T., S. M., and K. K. performed sampling. SH. K. wrote the first draft of the manuscript. 

J. J., M. T., H. B., and I. H. contributed to the conduction of the research process and data preparation. T. SH., MT. H., I. H., and M. A. managed the project, analyzed the data, and finalized the manuscript. All authors had full access to all the data in the study and take responsibility for the integrity of the data and the accuracy of the data analysis.

## Financial disclosure

No financial interests related to the material of this manuscript have been declared.
